# Can biological components predict short-term evolution in Autism Spectrum Disorders? A proof-of-concept study

**DOI:** 10.1186/s13052-016-0281-4

**Published:** 2016-07-22

**Authors:** Leonardo Emberti Gialloreti, Arianna Benvenuto, Barbara Battan, Francesca Benassi, Paolo Curatolo

**Affiliations:** Department of Biomedicine and Prevention, University of Rome “Tor Vergata”, via Montpellier 1, 00133 Rome, Italy; Department of Neuroscience, Pediatric Neurology Unit, University of Rome “Tor Vergata”, via Montpellier 1, 00133 Rome, Italy; Centre for Communication and Neurorehabilitation Research-CNAPP, via Marcantonio Boldetti 12, 00162 Rome, Italy

**Keywords:** Autism Spectrum Disorders, Developmental profiles, Autistic symptoms, Biological subtypes, Predicting factors

## Abstract

**Background:**

The clinical and pathogenetic heterogeneity of Autism Spectrum Disorders (ASD) limits our ability to predict its short- and long-term evolution. Aim of this naturalistic study was to observe the clinical evolution of very young children with ASD for 12 months after first diagnosis, in order to identify those children who might develop a more positive trajectory and understand how a wide range of biological, clinical and familial factors can influence prognosis.

**Methods:**

Ninety-two children were characterized in terms of family history, prenatal and perinatal variables, and clinical conditions. The sample was divided into four subgroups based on the association of 22 biological, clinical and family history variables. Developmental Quotient (DQ), determined using the Psychoeducational Profile Revised (PEP-R), and symptoms severity, measured by means of the Autism Diagnostic Observation Schedule (ADOS), were evaluated at baseline (T0) and after one year (T1), while receiving treatment as usual. Changes in DQ and ADOS between baseline and follow-up and differences in the short-term evolution of the four subgroups were analyzed.

**Results:**

At T1, 55.4 % of the children demonstrated some gains either of autistic symptomatology or of developmental skills. Mean ADOS score was 13.63 ± 3.67 at T0 and 10.85 ± 4.10 at T1 and mean DQ was 0.64 ± 0.14 at T0 and 0.66 ± 0.15 at T1. At follow-up, 33.7 % of the children showed an improvement in DQ and 37 % presented a less severe symptomatology, measured by means of ADOS. Overall, 15.2 % of the sample displayed major improvements both on developmental quotient and ADOS severity score; these children presented less EEG abnormalities and familial psychiatric disorders. The four subgroups, based on biological, clinical and familial variables, showed differing trends in terms of evolution.

**Conclusions:**

Categorizing very young children with ASD in terms of biological, clinical and familial variables can be instrumental in predicting short-term evolution. This exploratory study highlights the importance of a precise characterization and thorough analysis of interactions among biological and clinical variables, in order to predict the developmental evolution in children with ASD.

## Background

Autism Spectrum Disorders (ASD) are a group of heterogeneous neurodevelopmental conditions, characterized by persisting deficits in social communication and interaction in multiple contexts, as well as restricted, repetitive patterns of interests or activities [1]. ASD, affecting up to 1 every 100 school-age children, are considered to be an umbrella condition, characterized by specific genetic and biological underpinnings, significant heterogeneity in clinical presentation, different short- and long-term evolution and, possibly, diversified response to interventions [1, 2].

These disorders are determined by neurobiological abnormalities, as atypical brain development and alterations in synapses formation and connectivity [1]. Studies of genetic and epigenetic factors have suggested a polygenic nature of these conditions, but in most cases the exact nature of ASD’s aetiology remains elusive [2, 3]. Actually, in only 10–20 % of cases is possible to identify a syndromic form of autism related to a specific medical or genetic syndrome [3, 4].

Somatic and psychiatric comorbidity are highly represented in ASD, due to the underlying abnormalities in the biological pathways that can lead not only to alterations of the central nervous system, but also to systemic signs and symptoms, immune dysregulation, and sensory disturbances [5–7]. Although these symptoms are not directly related to the core features of ASD, they might have an important impact on the evolution and response to interventions [8].

The core symptoms of ASD become evident in early childhood. One of the major achievements in the last few years is the possibility of early diagnosis, already before 18–24 months of life, through the identification of specific signs and symptoms [1, 3]. Different studies have confirmed the importance of early detection and, therefore, early intervention for ASD children [9–11]. Some intervention approaches, mostly based on behavioural and developmental principles, eg Applied Behaviour Analysis (ABA) or the Early Start Denver Model, have been demonstrated to be efficacious in improving both autistic symptoms and cognitive, language and adaptive skills, at least in research settings and in some subgroups of subjects [12–14]. Family involvement has also been recommended as an efficacious component of early intervention [15, 16]. However, none of these approaches has been proved to substantially modify the diagnosis or natural course of ASD, which continue to be considered life-long disorders.

Actually, the clinical and pathogenetic heterogeneity of the disorder limits our ability to predict its short- and long-term evolution and may contribute to the current lack of effectiveness and to the variability in response to interventions that is observed across all evidence-based approaches [17, 18]. The heterogeneity and developmental nature of the disorder make it unlikely that one specific intervention will be best for all children with ASD and several researches point to the inadequacy of one single approach for all affected individuals [9, 19].

Some authors have attempted to identify biological subtypes of ASD, homogeneous in terms of clinical presentation and/or underlying pathogenesis, but few studies have analysed possible relationships between potential predicting factors and ASD evolution [20–22]. Baseline cognitive and language abilities are the most often reported correlates of positive developmental trajectories of children with ASD [10, 14]. Other studies have also identified more specific abilities associated with positive outcomes, including play skills, joint attention, imitation and low social avoidance; however, not all studies concur with these conclusions [17, 19]. In terms of clinical factors, several researches have demonstrated that children with any medical/genetic condition, including epilepsy, as well as children with a history of regression, had often the worst outcomes at long term follow-up [3, 8, 19]. It is plain that understanding how ASD unfold would be critical to better identify intervention goals and predict its evolution.

A multicentre autism consortium study has recently analysed the different autistic phenotypes by taking into account several developmental, clinical, and family history variables, including biological components [5, 7]. In the present study, we aimed to evaluate and understand if some specific biological and clinical factors could be helpful predictors of short-term evolution in a population of young children with ASD.

## Methods

### Design of the study

In this naturalistic longitudinal observational study, we distributed a sample of young children with ASD into four subgroups, according to the main characteristics of each child previously identified by means of principal components analysis performed and published by Sacco et al. [5]. Principal components analysis is a mathematical procedure that transforms a number of variables, retrieved from a large set of data, into a small number of uncorrelated variables called principal components. Its goal is to reduce the dimensionality of the data set, with a minimal loss of information, so to express the data in such a way as to highlight their similarities and differences. The first principal component accounts for as much of the variability in the data as possible, and each succeeding component accounts for as much of the remaining variability as possible [23].

In the study reported by Sacco and co-workers, principal component analysis has been used, in a sample of people with ASD, to try to reduce 22 possible outcome predictors into a small number of components, while minimizing the loss of information. The 22 biological, clinical, and family history variables included in the analysis, which eventually identified four subgroups, are presented in Table 1. As a result of the principal component analysis, each subgroup was characterized by a prevalent, though not unique, feature: (I) disruption of the sleep–wake cycle associated with hyperactivity and sensory abnormalities (CS), (II) immune dysregulation, associated with prenatal obstetric complications (ID), (III) generalized neurodevelopmental delay (ND), and (IV) stereotypies and abnormal early social behaviours (SB) [5]. In our study we aimed to evaluate if these four principal components were able to predict the outcome of a population of young ASD children 12 months after their first diagnosis.

**Table 1:** Biological, clinical, and family history variables included in the analysis, which eventually identified the four subgroups used in the study (from Sacco et al. 2010)

Developmental and clinical variables	Biological variables	Family history variables
Age at non-verbal language development	History of allergies	Pregnancy duration
Age at verbal language development	History of regression	History of obstetric complications or recurrent spontaneous abortions in the mother
Level of verbal language development	History of obstetric complications at or immediately after birth	History of any allergic and/or immune disease in the family
Age at walking	History of any infectious disease at autism onset	History of tumors in the family
Age at acquisition of bladder control at night	History of sleep disorders	History of any neurological or psychiatric disorders in the family
Age at first social smile	Presence of muscle hypotonia at neurological examination	
Motor, verbal or vocal stereotypies	EEG pattern (evaluated at T0)	
Self-aggressive or self-injurious behaviour	Pain sensitivity (reported by parents)	
Hyperactivity		

In order to possibly enlarge the informative value of these components, we added also data about the co-presence of other clinical/medical conditions of the child or of his/her first-degree relatives. We chose a pretest-posttest design in order to follow the development of the sample and to determine potential correlations between some specific factors and short-term evolution. All patients received their first diagnosis and were evaluated at baseline (T0) in the outpatient clinic of the Pediatric Neurology Unit of the University of Rome “Tor Vergata”, in order to determine clinical characteristics, developmental level, and symptom severity. Participants were re-examined one year after their first evaluation (T1).

### Participants

One hundred two children with a diagnosis of ASD were consecutively recruited in the study. To be included in the present study, children had to receive an ASD diagnosis by two independent child neurologists of our research team, according to the ‘Italian guidelines on ASD management’ [24]. Enrolment took place from January to July 2014, a time in which DSM-V was not yet available in Italy. For this reason, we have used the diagnostic criteria based on the *Diagnostic and Statistical Manual of Mental Disorders, Fourth Edition* (DSM-IV) for the diagnosis of Autism Spectrum Disorders and we have included Autistic Disorder (AD), Pervasive Developmental Disorder Not Otherwise Specified (PDD-NOS) and Asperger Syndrome (AS). We have used the DSM-IV criteria also at the time of follow up, in order to guarantee a homogeneous diagnostic classification of the sample.

Exclusion criteria of the study included (1) neurodevelopmental disorders of known aetiology (eg Fragile X Syndrome, Tuberous Sclerosis, or known chromosomal abnormalities or metabolic disorders), (2) significant sensory or motor impairment, and (3) serious chronic diseases. We did not consider epilepsy as an exclusion criterion; nevertheless, no child had seizures or suffered from epilepsy at the time of enrolment. Furthermore, at T0 no child was under pharmacological treatment.

Out of the 102 considered children, 5 were excluded because they were affected by medical conditions with a significant sensory or motor impairment that could influence the children’s ability to complete the whole diagnostic assessment; specifically, four of them were excluded for the presence of significant hearing and/or vision impairments, and one child was excluded for the presence of a major motor disability due to Duchenne dystrophy. Other 5 children were identified as potentially eligible, but their families did not agree to participate in the study. Consequently, 92 children (84 boys and 8 girls) were eventually enrolled in the study.

### Clinical assessment

All children were examined both at baseline (T0) and at follow-up (T1). In addition, at T0 all patients underwent a defined medical workup, including neurological examination, awake/sleep EEG, as well as height, weight and head circumference measurement. The retrieved data were completed by examining their medical records. Clinical assessment of the children included two different areas: (1) The administration of developmental and symptomatological tests and (2) the collection of the child’s medical history. The diagnostic protocol included the administration of the Psychoeducational Profile Revised (PEP-R) for the identification of both global developmental level and specific profiles of the six PEP-R sub-items: cognitive abilities, expressive language, receptive language, gross-motor abilities, fine-motor abilities, and visual-motor imitation [25]. The PEP-R is widely used in clinical settings and its utility has been extended to research work to describe clinical features and short-term evolution of children with ASD. Although the PEP-R was not originally designed to determine general level of intellectual functioning, several studies have suggested that this test demonstrates a good internal consistency and inter-rater reliability, provides a good estimate of cognitive abilities in young children with autism and/or other disabilities, and is a sensitive pre-post measure for the evaluation of clinical evolution [26, 27].

The Autism Diagnostic Observation Schedule (ADOS) was administered to confirm diagnosis and to determine the severity of autistic symptoms [28]. In addition, the Autism Diagnostic Interview-Revised (ADI-R) was also used to confirm the diagnosis at T0 [29].

Two different child neurologists of our research team, which are expert in the diagnostic management of young children with ASD, were involved in the administration of the various parts of the diagnostic protocol. Both evaluators were blind to the study hypotheses. One assessor administered the PEP-R at T0 to determine the Developmental Quotient/Intelligence Quotient (DQ) and the developmental profile in the six PEP-R sub-items. One year later, PEP-R was re-administered by the same assessor in order to re-establish the child’s DQ in all six areas of the scale.

The ADOS was administered both at T0 and T1 by another assessor of our research team, which had completed the ADOS training and is in possession of an ADOS research certification. According to the child’s language level, the examiner used module 1 (minimal to no language) for the evaluation of 80 children, and module 2 (non-echoed phrase speech) for the other 12 children.

The whole sample was tested with the two instruments at T0 and T1. For the PEP-R scale, the entire protocol was administered in one or two sessions, depending on the cooperation of the child. All children were able to complete their level of functioning diagnostic assessment. Moreover, the two assessors collected the clinical data of medical history of the enrolled children and supervised their medical workup.

The recording of the medical history of the children encompassed family history variables (parental and first-degree relative diseases, history of obstetric complications or recurrent spontaneous abortions in the mother, neuropsychological disorders in parents and/or siblings), prenatal or perinatal variables (pregnancy course, pharmacological treatment or infections during gestation, birth weight, perinatal complications), and information about the presence of clinical conditions in the affected individuals, ie, history of regression (defined as loss of more than five spoken words used communicatively), sleep disorders, history of allergies, and gastrointestinal disorders. The socio-economic status of the family was categorized into lower-, middle-, and upper-class, based on the family annual revenue share and parental level of education, in accordance with the criteria of the Italian Statistical Bureau (ISTAT) [30].

### Measurement of the changes over time

To evaluate the evolution after one year (T1) we used two measures: ‘Change of DQ rate’ and ‘change of ADOS diagnostic scores’. We analysed the two measures both independently and related to one another.

Regarding DQ values, we set the threshold for change in developmental level as an increase/decrease of 5 % DQ score and then we generated three groups: ‘DQ improved group’ for those patients whose DQ level raised from T0 to T1 by at least 5 %; ‘DQ worsened group’ for those patients whose DQ level decreased by at least 5 %; and ‘DQ stable group’ for those patients with a DQ change of less than 5 %.

Regarding changes in ADOS scores, subjects have been dichotomized into two groups. Those subjects, whose ADOS composite score improved between T0 and T1 were included in the “ADOS Improvement group”. The others were included in the “ADOS No-improvement group”. We used also the Calibrated Severity Score (CSS) and the change of the ADOS classification category as two secondary measures of quantitative and qualitative symptomatological changes. The CSS is a standardized score of the relative severity of autism-specific behaviours; this measure has been created by Gotham and co-workers to compare autistic symptoms within and across individuals of different ages [31]. We included CSS among the measures of symptomatological change because it is less influenced by age and developmental level than the ADOS raw totals.

### Interventions

Aim of this study was not to investigate or to compare the efficacy of different interventions. Nevertheless, all the included children underwent some form of intervention during the follow-up period. As it is common in the Italian public health system, interventions did not take place at our centre, but families have been referred to community rehabilitation centres. The modality of community-based treatment follows the ‘Italian guidelines on ASD management’ [24]. In Italy, treatment as usual is composed of specific interventions performed by child neuropsychiatric services (CNS) and of school inclusion activities. In terms of school inclusion strategies, the Italian programs are based on a co-teaching model in which students with and without disabilities work together in the same classroom; children with disabilities are also trained by a “support teacher” and benefit from an individualized educational plan [11].

Intervention models offered by the local health services include sessions of individual psychomotricity and/or speech and/or psycho-educative therapy. Each child had an individualized treatment plan that incorporated a range of objectives, dependent on the child’s level of functioning, but, as a matter of fact, it is mainly based on staff expertise rather than standardized treatment protocols. All interventions were delivered by therapists and implemented also in familial and school settings.

Treatments as usual can be located in the behavioural/developmental continuum, from highly structured behavioural approaches, guided by a therapist, to approaches that follow the interests of the child in a naturalistic setting; this model is always based on individualization to each infant’s developmental profile and focused on a broad range of learning targets. In several cases, treatment as usual includes a number of hours of parent coaching or parent involvement during the child–therapist work sessions, in order to support parental sensitivity to child cues and to implement intervention targets with their child during and outside of the sessions [15, 32].

In our study, mean age at start of treatment as usual was 38.1 ± 7.8 months. The participating children received treatment as usual for a mean of 10.7 h per week, including hours of individual sessions and school inclusion.

### Statistical analysis

Changes between baseline and follow-up were evaluated with paired samples t tests or chi-square tests. Differences between groups were analysed by means of t-tests for independent samples, ANOVA models, Pearson’s Chi-squared tests, or Fishers Exact tests (for small groups). Spearman’s rho correlation coefficients were used to examine associations between quantitative measures. The effect sizes were estimated by means of Cohen’s d. We always applied a significance level of 0.05, Bonferroni-corrected for multiple comparisons. We decided to use the more stringent Bonferroni correction given the high number of statistical comparisons included in the study to guard against Type I errors. Results are reported as means ± SDs. All analyses were performed using R Language and Environment for Statistical Computing programme (http://www.R-project.org; accessed April 2016) [33].

### Ethics

All performed procedures were in accordance with the ethical standards of the institutional and national research committee and with the 1964 Helsinki declaration and its later amendments [34]. Informed consent to the participation in the research and to the publication of patients’ data was obtained from the parents or legal guardian of all individuals included in the study (all children were under age 16). Study procedures were approved by our research ethics committee.

## Results

### Distribution of clinical features and medical variables in the whole sample

#### Clinical features

Ninety-two children (84 boys and 8 girls), whose age at baseline ranged from 18 to 50 months, were enrolled in the study. At T0, mean age of the participating children was 36.9 ± 7.6 months. Female/male ratio was 1:9. In terms of developmental characteristics, measured by means of the PEP-R, mean developmental age was 23.4 ± 6.8 months and mean developmental quotient (DQ) was 0.64 ± 0.14. Concerning language skills at T0, 58.7 % of the children were nonverbal, while 31.5 % used single words, and 9.8 % used phrases. In terms of symptomatological severity, according to ADOS cut-offs at baseline, 49 children have been identified as PDD-NOS, 39 as AD, and 4 as non-autism. After thorough analysis and discussion between the two child neurologists, the latter four children have been nevertheless included in the study as they had been clinically diagnosed as PDD-NOS, according to the DSM-IV criteria and the Italian guidelines. Table 2 summarizes the basic demographic and clinical features of the sample.

**Table 2:** Basic characteristics of the sample

	*N* (Percent)
Females	8 (8.7 %)
Males	84 (91.3 %)
	Mean ± SD
Chronological age (months) at T0	36.9 ± 7.6
Chronological age (months) at T1	49.3 ± 7.7
Diagnosis based on ADOS cut-offs at T0	*N* (Percent)
Autism Spectrum Disorder	49 (53.3 %)
Autism	39 (42.4 %)
Non Autism	4 (4.3 %)
	Mean ± SD
Developmental age (months) measured by PEP-R at T0	23.4 ± 6.8
Developmental age (months) measured by PEP-R at T1	32.6 ± 9.0

*N* = 92, *ADOS* Autism Diagnostic Observation Schedule, *PEP-R* Psychoeducational Profile Revised

#### Clinical and medical history variables

Thirty-six children (39.1 %) had a history of allergic disorders, 23 (25.0 %) had a positive history for regression, 15 (16.3 %) presented macrosomy, 9 (9.8 %) had EEG alterations, 27 (29.3 %) presented with sleep problems in their case history.

No patient began a pharmacological treatment or developed seizures or any further neurological condition during the follow-up time.

#### Family factors

In terms of familial risk factors, 56.5 % of the families reported neurological or psychiatric disorders in one or more first-degree relatives. Twenty-five families (27.2 %) were categorized as upper-class, 57 (62 %) as middle-class and 10 (10.8 %) as lower-class.

#### Individual clinical features at T0 and T1

Mean chronological age of the participating children was 36.9 ± 7.6 at baseline (T0) and 49.3 ± 7.7 at follow-up (T1). Mean developmental age, tested using the PEP-R scale, was 23.4 ± 6.8 at T0 and 32.6 ± 9.0 at T1. Mean developmental quotient (DQ) was 0.64 ± 0.14 at T0 and 0.66 ± 0.15 at T1. Mean ADOS composite score was 13.63 ± 3.67 at T0 (Median: 14) and 10.85 ± 4.10 at T1 (Median: 11). The overall difference of ADOS score between T0 and T1 was -2.78 ± 3.98, ie, an average improvement of 11.6 %. After one year of follow-up we observed also a significant score decrease in terms of CSS (*p* < 0.001). Biological, clinical, and family history variables included in the analysis, which eventually identified the four subgroups used in the study, have been previously described in Table 1.

### Short-term evolution of the whole sample

Mean duration of follow-up was 12.4 ± 2.3 months.

#### Change in Developmental Quotient

At T1, 31 children (33.7 %) have been included in the ‘DQ improved group’, 41 in the ‘DQ stable group’ (44.6 %), and 20 (21.7 %) in the ‘DQ worsened group’. Overall, DQ values showed a statistically significant increase (*p* < 0.01) with respect to baseline measures. The analysis of PEP-R sub-quotients showed a considerable change especially in the cognitive (*p* < 0.0001), receptive (*p* < 0.05), and expressive language (*p* < 0.01) areas; conversely, visual-motor imitation, gross-motor and fine-motor areas remained substantially stationary.

#### Changes in ADOS diagnostic scores

At T1, ADOS improved in 69 (75.0 %) children (Improvers), whereas it was the same or worse in 23 (25.0 %) children (Non-Improvers). Interestingly, the “ADOS Improvers group” had a significantly (*p* < 0.004; Cohen’s d: 0.71) higher ADOS score at baseline (14.24 ± 3.58) compared to the “ADOS Non-Improvers group” (11.74 ± 3.35). On the other hand, ADOS score at T1 was 9.78 ± 3.85 among “ADOS Improvers” and 14.04 ± 3.10 among “ADOS Non-Improvers” (*p* < 0.0001; Cohen’s d: 1.11). On average, “ADOS Improvers” ameliorated by 4.48 ± 2.89 points, compared to the “ADOS Non-Improvers” who worsened by 2.30 ± 1.94 points. In other words, those who fared worse at baseline tended to improve more. Analogously, also CSS at T0 (5.51 ± 1.51 vs. 4.83 ± 1.03; *p* < 0.05; Cohen’s d: 0.45) and at T1 (4.10 ± 1.43 vs. 5.74 ± 0.62; *p* < 0.0001; Cohen’s d: 1.15) was significantly different between “ADOS Improvers” and “ADOS Non-Improvers”. In order to analyse ASD severity modifications, we used change in ADOS categories (from ‘autism’ to ‘autism spectrum’ or ‘non-autism’, and from ‘autism spectrum’ to ‘non autism’). At T1, 34 children (37.0 %) modified their ADOS classification in a less severe category; on the other hand, 42 children (45.6 %) remained stable and 16 (17.4 %) were included in a more severe category (from ‘non autism’ to ‘autism spectrum’ or ‘autism’; from ‘autism spectrum’ to ‘autism’). Further results are presented in Table 3.

**Table 3:** Clinical measures of the whole sample at T0 and T1, and number of children who improved in the different domains

	M ± SD		
	T0	T1	*p*
ADOS composite	13.63 ± 3.67	10.85 ± 4.10	**<0.0001**
A	4.72 ± 1.74	3.39 ± 1.75	**<0.001**
B	8.91 ± 2.27	7.46 ± 2.79	**<0.0001**
C	2.86 ± 1.11	2.39 ± 1.28	**<0.01**
D	3.71 ± 1.56	3.17 ± 1.75	**<0.01**
CSS	5.34 ± 1.44	4.51 ± 1.46	**<0.0001**
DQ	0.64 ± 0.14	0.66 ± 0.15	**<0.01**
	DQ sub-quotients:		
Cognitive abilities	0.72 ± 0.19	0.77 ± 0.21	**<0.0001**
Expressive language	0.44 ± 0.16	0.47 ± 0.19	**<0.05**
Receptive language	0.52 ± 0.18	0.57 ± 0.23	**<0.01**
Fine motor abilities	0.73 ± 0.15	0.73 ± 0.17	=0.915
Gross motor abilities	0.78 ± 0.16	0.78 ± 0.17	=0.799
Visual motor imitation	0.68 ± 0.15	0.68 ± 0.14	=0.964
	Number (Percent)		
Improvement in both ADOS classification and DQ level	14 (15.2 %)		
Improvement in ADOS classification	34 (37.0 %)		
Improvement in DQ level	31 (33.7)%		
Improvement in cognitive abilities	47 (51.1 %)		
Improvement in expressive language	40 (43.5 %)		
Improvement in receptive language	35 (38.1 %)		
Improvement in fine-motor abilities	30 (32.6 %)		
Improvement in gross motor abilities	35 (38.1 %)		
Improvement in visual-motor imitation	29 (31.5 %)		

*N* = 92; *ADOS* Autism Diagnostic Observation Schedule, *CSS* Calibrate Severity Score, *DQ* Developmental Quotient, Statistically significant results are in **bold**

#### Correlation between changes in ADOS scores and DQ

No significant differences have been observed between “ADOS Improvers” and “ADOS Non-Improvers” in terms of Global DQ at T0 (0.63 ± 0.14 vs. 0.62 ± 0.12; Cohen’s d: 0.07) or at T1 (0.67 ± 0.16 vs. 0.64 ± 0.14; Cohen’s d: 0.20). No other statistically significant differences were demonstrated by the analysis of the correlation between the two measures.

According to the definition of positive evolution, about 55.4 % of the participants showed a clinical improvement at follow-up, although it appeared to be partial and linked only to one measure (either ADOS or DQ) in 40.2 % of our children. There was no statistically significant association between severity decrease, measured by means of ADOS, and improvement of DQ levels. This means that not all children that showed a positive change in one measure improved necessarily also in the other one.

### Short-term evolution of the “Major Improvers” group

Fourteen children (15.2 %) displayed a significant improvement both of developmental level and symptoms severity. We have labelled this subgroup as ‘Major Improvers’ and we conducted a more detailed analysis of these patients, in order to understand if this particular subclass showed different features and/or phenotypes, which might explain a better evolution at follow-up.

Considering individual pre-treatment features, the level of verbal-language skills was higher, though not significantly, for children in the ‘Major Improvers’ group, compared to the others (35.7 % of children without verbal language vs. 65.9 %).

In terms of associated medical variables, the analysis found that ‘Major Improvers’, compared to those who worsened both at ADOS and DQ, presented with a positive- although non statistically significant - tendency not to have EEG abnormalities (0 % vs. 12.2 %), not to have allergic disorders (28.6 % vs. 44.0 %), not to belong to a family with low socioeconomic status (0 % vs. 14.6 %).

With respect to familial factors, the occurrence of neurological or psychiatric disorders, even if not statistically significant, was lower in the ‘Major Improvers’ families (50.0 %) than in those who improved neither in symptomatology nor in developmental quotient (63.4 %).

### Biological components and associated clinical predictors in the four subgroups

Lastly, we stratified all participants based on the four previously identified subgroups and evaluated potential associations between their evolution and belonging to a specific subgroup. Twenty-four children (26.1 %) were classified in the CS subgroup, 31 (33.7 %) in the ID subgroup, 9 (9.8 %) in the ND subgroup, and 28 (30.4 %) in the SB subgroup. Table 4 provides an overview of the medical and clinical variables of the four subgroups. In terms of demographic, medical, and/or co-morbid conditions, we did not observe significant differences among the four subgroups, except for those already considered as attribution criteria to a specific subgroup (eg a significant difference in sleep disorders for the ‘circadian and sensory dysfunction’ CS subgroup, or in pre/perinatal complications for the ‘immune dysfunction’ ID subgroup).

**Table 4:** Medical complaints and familial factors of the four subgroups at T0

	CS (*N* = 24)	ID (*N* = 31)	ND (*N* = 9)	SB (*N* = 28)	
	Number (%)	Number (%)	Number (%)	Number (%)	*p*
EEG abnormalities	3/24 (12.5 %)	2/31 (6.5 %)	2/9 (22.2 %)	2/28 (7.1 %)	=0.495
History of gastrointestinal disorders	13/24 (54.2 %)	18/31 (58.1 %)	5/9 (55.6 %)	7/28 (25.0 %)	**<0.05**
History of allergies	14/24 (58.3 %)	15/31 (48.4 %)	1/9 (11.1 %)	6/28 (21.4 %)	**<0.01**
History of sleep disorders	12/24 (50.0 %)	9/31 (29.0 %)	3/9 (33.3 %)	3/28 (10.7 %)	**<0.05**
Prenatal, perinatak or postnatal complications	8/24 (33.3 %)	25/31 (80.6 %)	4/9 (44.4 %)	8/28 (28.6 %)	**<0.001**
Macrocrania	1/24 (4.2 %)	7/31 (22.6 %)	3/9 (33.3 %)	4/28 (14.3 %)	=0.107
History of regression	8/24 (33.3 %)	5/31 (16.1 %)	4/9 (44.4 %)	6/28 (21.4 %)	=0.243
Family history of psycho-neurological disorders	16/24 (66.7 %)	17/31 (54.8 %)	3/9 (33.3 %)	16/28 (57.1 %)	=0.399
Family history of tumors	3/24 (12.5 %)	5/31 (16.1 %)	2/9 (22.2 %)	3/28 (10.7 %)	=0.817

*CS* Circadian and sensory dysfunction subgroup, *ID* Immune dysfunction subgroup, *ND* Neurodevelopmental delay subgroup, *SB* Stereotypic behaviour subgroup; Statistically significant results are in **bold**

#### DQ and ADOS scores in the four subgroups at T0

In terms of global DQ at baseline, lower DQ values were significantly associated with the inclusion in the ND subgroup (*p* < 0.0001). Specifically, mean DQ level at T0 was 0.61 ± 0.12, 0.62 ± 0.13, 0.73 ± 0.12, and 0.50 ± 0.09 in the CS, ID, SB, and ND subgroups, respectively. Conversely, the individual core of ASD features, measured at T0 by means of the ADOS score, did not show statistically significant differences between the four subgroups (13.79 ± 3.61, 13.84 ± 3.83, 12.50 ± 3.28, and 16.00 ± 3.64, in the CS, ID, SB, and ND subgroups, respectively). Nevertheless, there is a tendency of the SB subgroup to manifest a lower ADOS composite score, compared to the three other subgroups. On the other hand, the ND subgroup was characterized by a greater symptomatological severity, albeit also this difference was not statistically significant. There were no significant differences between the four subgroups on any familial variable.

#### Change of DQ and ADOS scores in the four subgroups between T0 and T1

**Fig. 1:**
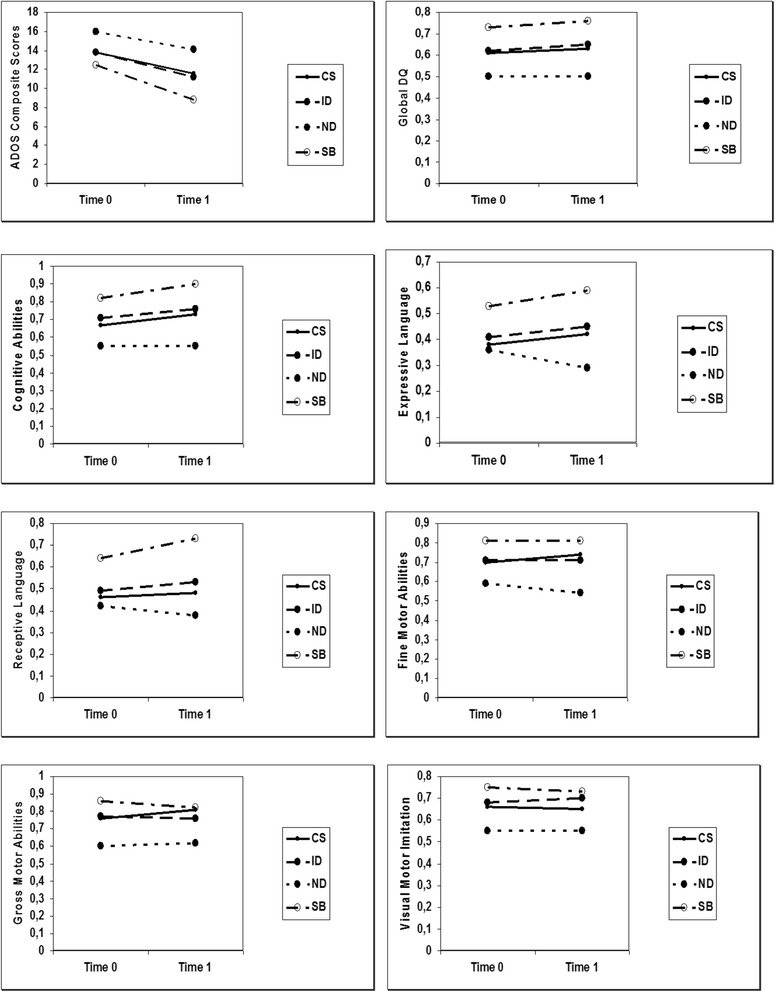
Overview of the trends of the four subgroups from T0 to T1. The total rate of the ADOS composite score differed significantly at T0 and T1 for the CS, ID, and SB subgroups. For the DQ values, none of the four subgroups showed a statistically significant change between T0 and T1. The independent analysis of PEP-R sub-areas revealed that the CS subgroup showed a significant improvement in the cognitive area and the SB subgroup presented a higher expressive and receptive language level. CS: Circadian and sensory dysfunction subgroup; ID: Immune dysfunction subgroup; ND: Neurodevelopmental delay subgroup; SB: Stereotypic behaviour subgroup; ADOS: Autism Diagnostic Observation Schedule

With respect to modifications of the DQ rate and ADOS category, we found a few statistically significant associations between a positive evolution and belonging to a specific biological subgroup (see Table 5 and Fig. 1 for details).

**Table 5:** Clinical measures of the evolution for the four subgroups at T0 and T1

	CS (*N* = 24)			ID (*N* = 31)			ND (*N* = 9)			SB (*N* = 28)		
	T0	T1	*p*	T0	T1	*p*	T0	T1	*p*	T0	T1	*p*
ADOS composite	13.79 ± 3.61	11.58 ± 3.82	**<0.05**	13.84 ± 3.83	11.23 ± 4.01	**<0.0001**	16.00 ± 3.64	14.11 ± 3.55	=0.120	12.50 ± 3.28	8.75 ± 4.11	**<0.001**
A	4.88 ± 1.85	3.75 ± 1.65	**<0.05**	4.90 ± 1.68	3.35 ± 1.58	**<0.0001**	5.44 ± 2.01	4.78 ± 1.48	=0.373	4.14 ± 1.53	2.68 ± 1.83	**<0.001**
B	8.92 ± 2.19	7.83 ± 2.58	=0.083	8.94 ± 2.41	7.87 ± 2.78	**<0.01**	10.56 ± 1.74	9.33 ± 2.29	=0.055	8.36 ± 2.18	6.07 ± 2.64	**<0.01**
C	2.67 ± 1.05	2.29 ± 1.27	=0.266	3.13 ± 1.09	2.48 ± 1.31	**<0.01**	3.44 ± 0.88	3.22 ± 1.30	=0.622	2.54 ± 1.14	2.11 ± 1.20	=0.089
D	3.92 ± 1.44	2.88 ± 1.62	**<0.05**	3.55 ± 1.67	3.52 ± 1.57	=0.903	4.44 ± 1.88	4.33 ± 2.06	=0.346	3.46 ± 1.40	2.68 ± 1.79	**<0.01**
CSS	5.58 ± 1.61	4.75 ± 1.36	**<0.05**	5.23 ± 1.63	4.68 ± 1.42	**<0.05**	5.56 ± 1.01	5.12 ± 1.05	=0.220	5.18 ± 1.19	3.93 ± 1.56	**<0.01**
Global DQ	0.61 ± 0.12	0.63 ± 0.11	=0.070	0.62 ± 0.13	0.65 ± 0.16	=0.190	0.50 ± 0.09	0.50 ± 0.07	=0.907	0.73 ± 0.12	0.76 ± 0.14	=0.060
Cognitive abilities	0.67 ± 0.17	0.73 ± 0.16	**<0.05**	0.71 ± 0.20	0.76 ± 0.20	=0.087	0.55 ± 0.10	0.55 ± 0.14	=0.855	0.82 ± 0.16	0.90 ± 0.19	=0.055
Expressive language	0.38 ± 0.08	0.42 ± 0.14	=0.163	0.41 ± 0.12	0.45 ± 0.18	=0.179	0.36 ± 0.08	0.29 ± 0.08	**<0.05**	0.53 ± 0.23	0.59 ± 0.21	**<0.05**
Receptive language	0.46 ± 0.15	0.48 ± 0.12	=0.342	0.49 ± 0.14	0.53 ± 0.21	=0.156	0.42 ± 0.08	0.38 ± 0.10	=0.110	0.64 ± 0.20	0.73 ± 0.26	**<0.05**
Fine motor abilities	0.70 ± 0.16	0.74 ± 0.14	=0.200	0.71 ± 0.14	0.71 ± 0.17	=0.733	0.59 ± 0.12	0.54 ± 0.07	=0.097	0.81 ± 0.12	0.81 ± 0.16	=0.995
Gross motor abilities	0.76 ± 0.18	0.81 ± 0.19	=0.332	0.77 ± 0.15	0.76 ± 0.16	=0.629	0.60 ± 0.14	0.62 ± 0.13	=0.721	0.86 ± 0.13	0.82 ± 0.17	=0.112
Visual motor imitation	0.66 ± 0.16	0.65 ± 0.10	=0.783	0.68 ± 0.14	0.70 ± 0.15	=0.448	0.55 ± 0.14	0.55 ± 0.09	=0.914	0.75 ± 0.11	0.73 ± 0.13	=0.586

*CS* Circadian and sensory dysfunction subgroup, *ID* Immune dysfunction subgroup, *ND* Neurodevelopmental delay subgroup, *SB* Stereotypic behaviour subgroup, *ADOS* Autism Diagnostic Observation Schedule, *CSS* Calibrate Severity Score, *DQ* Developmental Quotient. Statistically significant results are in **bold**

Concerning global DQ values, none of the four subgroups showed a statistically significant change between T0 and T1. Overall, the independent analysis of PEP-R sub-areas revealed a substantial stability of all developmental skills, however, the CS subgroup showed a significant improvement in the cognitive area (*p* < 0.05) and the SB subgroup presented with a statistically significant higher expressive and receptive language level (*p* < 0.05).

Also the total rate of the ADOS composite score differed significantly between T0 and T1 for the CS, ID and SB subgroups, suggesting an improvement of symptoms severity. Particularly, the CS subgroup displayed a significantly lower score in ‘Language and Communication’ and ‘Stereotyped Behaviours and Restricted Interest’; the SB subgroup showed a marked improvement in all sub-areas, except in the ‘Play’ item, while the ID subgroup ameliorated symptomatological levels in ‘Language and Communication’, ‘Social Interaction’ and ‘Play’, but not in ‘Stereotyped behaviours and Restricted Interest’. On the other hand, the ND subgroup did not reach any significant improvement in any ADOS sub-area.

## Discussion

In this study, we classified 92 young children with ASD into four subgroups, according to their biological, medical and family history characteristics; we observed their evolution for 12 months after first diagnosis, in order to identify those children who might develop a more positive trajectory and understand how a wide range of biological, medical and clinical factors might influence evolution.

After one year of follow-up, more than half of the study participants obtained some gains either in terms of autism severity or of developmental skills. Moreover, our study demonstrated that even in such a brief period of observation 15 % of the sample, defined as ‘Major Improvers’, showed significant advances on standard scores of autism severity, adaptive behaviours and developmental level. The ‘Major Improvers’ showed a positive tendency, although non-statistically significant, to present less EEG abnormalities and less psychiatric disorders in their family history compared to the ‘Non Improvers group’. Notwithstanding the lack of a statistically significant improvement, we believe that it is meaningful to describe such a tendency, as previous studies on developmental trajectories have revealed the existence of a particular category of children, identified as “bloomers” or “accelerated”, characterized by a quick improvement over time, possibly representing a sort of neurobiological and genetically protected category [3, 35, 36]. On the other hand, it has been demonstrated that autistic children with some medical conditions, history of seizures, and severe intellectual disability are more likely not to improve over time [19, 37].

The variability in terms of developmental evolution observed in different studies probably reflects the heterogeneity of ASD samples. In our study the univariate analysis of the different variables lead only to a few statistically significant results. As a matter of fact, given the complexity of the biological underpinning of ASD, current knowledge of factors affecting its evolution is limited, and single behavioural or biological predictors may not be sufficient to correctly foretell upshots [38, 39].

For this reason, we tried to identify some biological subgroups as well, so to outline if the combination of different biological components, and not only a single factor, might be associated with a specific developmental course. We therefore identified, by means of principal component analysis, four subgroups characterized by a prevalent - though not unique - feature, based on the association of 22 biological, clinical and family history variables [5]. In this way, each child was included in one of the four subgroups. In terms of the potential correlation between belonging to a subgroup and having a specific developmental profile or global DQ values, we observed that the four subgroups showed at follow-up a substantial stability of all developmental skills and DQ scores; however, we observed also that belonging to a subgroup was associated with a positive trend in some specific skill areas: the ‘circadian and sensory dysfunction’ subgroup showed a significant improvement in the cognitive area, while the ‘stereotypic behaviour’ subgroup presented with a higher expressive and receptive language level at follow-up. Also in terms of symptomatological changes, the ‘circadian and sensory dysfunction’ subgroup fared better in language, communication, and stereotyped behaviours, while the ‘stereotypic behaviour’ subgroup improved in all these sub-areas. The ‘immune dysfunction’ subgroup ameliorated in language and social interaction, but not in terms of stereotyped behaviours. Overall, we observed positive trends, particularly in the ‘stereotypic behaviour’ subgroup, and negative trends mainly in the ‘neurodevelopmental delay’ subgroup. These observations – even though exploratory and preliminary - are, in our knowledge, among the first ones to suggest using multiple biological, clinical and familial factors as predictors of evolution.

There are several limitations of this study. Firstly, we could not compare the eclectic, non-standardized intervention approaches, which characterize treatment as usual in Italy. Hence, we were unable to make any hypothesis regarding which specific treatment ingredient was leading to a better outcome. In addition, the heterogeneity of interventions received by the participants could have affected the interpretation of the results. In fact, we could not infer what proportion of the evolution should be attributed to the intrinsic child’s characteristics or environmental factors, rather than to the intervention. In this view, the naturalistic approaches, contrary to RCTs, are not able to address the question of the effectiveness of one specific intervention. However, naturalistic studies allow evaluating real life settings [11, 18]. Secondly, we analysed a large number of factors in a fairly limited sample. Furthermore, as several of the studied factors are related to each other and, thus, can act both as independent variables and/or mediators, it may be difficult to address causality. Moreover, the intrinsic heterogeneity of the sample and the underrepresentation of females may have affected the results.

Although this study highlighted some promising biological predicting factors, it did not consider all potential factors. Therefore, the present study is exploratory; a starting point to more accurate predictions. Our results should be verified and confirmed by larger prospective studies. It should also be mentioned that, although the inter-rater reliability of the standardized tests are considered to be high and their objectivity in a naturalistic clinical setting is well established, biases due to differing raters cannot be entirely excluded [18, 39]. Lastly, we observed evolution after one year of follow-up, hence, our conclusions are limited only to short-term changes, and cannot be automatically transposed to long-term ones. Longer follow-up periods are needed to enlarge the applicability of the results.

Notwithstanding these limitations, we believe that our findings might be helpful to set up instruments that take into account the considerable phenotypic variability of ASD to predict the evolution of this disorder. Our study highlights the importance of taking into account possible predicting factors not as single variables, but always considering their interactions.

## Conclusion

There is a paucity of studies that investigate biological components associated with the developmental course of young children with ASD. The needs of children with ASD are complex and this is reflected in the diversified evolutions, as well as measurement tools used to collect evidence about the child’s progress. The heterogeneity of evolutions in children with ASD compounds the difficulty in understanding whether different aetiologies are associated with differing phenotypic expressions and short or long-term course. Moreover, current knowledge of the factors associated with individual differences in development is often limited to the analysis of specific behavioural abilities and clinical studies are not designed to consider the contribution of other potential risk factors, like the biological ones. The development of a more detailed research methodology is mandatory to delineate new specific clinical profiles or ASD subgroups and consequently to better understand the critical difference between individual trajectories and group level evolutions. In this view, the research on predictors of development, including biological components, should be on top of the ASD research agenda.

In our opinion, the results of the present study - among the first ones to consider also biological predictors - emphasize that taking into account a large number of biological, clinical and familial factors can be useful to better predict evolution. Adding other factors, not taken into account by our exploratory study, will probably further enhance our ability to foretell a child’s evolution. In any case, our proof-of-concept study has highlighted that only by analysing the multiple interactions of different factors one can improve its capability to predict ASD evolution at individual level. Indeed, in future we should increasingly look at the clinical characteristics of the children with ASD also from the perspective of their possible interconnections with the many-sided biological, genetic and environmental factors.

## Abbreviations

AD, Autistic Disorder; ADI-R, Autism Diagnostic Interview Revised; ADOS, Autism Diagnostic Observation Schedule; ASD, Autism Spectrum Disorders; CNS, child neuropsychiatric services; CS, circadian and sensory dysfunction; CSS, Calibrated Severity Score; DQ, Developmental Quotient; DSM-IV, Diagnostic and Statistical Manual of Mental Disorders, Fourth Edition; ID, immune dysfunction; IQ, Intelligence Quotient; ND, neurodevelopmental delay; PDD-NOS, Pervasive Developmental Disorder Not Otherwise Specified; PEP-R, Psychoeducational Profile Revised; SB, stereotypic behaviours
